# The effect of metformin treatment *in vivo* on acute and long-term energy metabolism and progesterone production *in vitro* by granulosa cells from women with polycystic ovary syndrome

**DOI:** 10.1093/humrep/deu187

**Published:** 2014-08-19

**Authors:** D. Maruthini, S.E. Harris, J.H. Barth, A.H. Balen, B.K. Campbell, H.M. Picton

**Affiliations:** 1 The Leeds Centrefor Reproductive Medicine, Seacroft Hospital, York Road, LeedsLS14 6UH, UK; 2 Division of Reproduction and Early Development, Leeds Institute for Genetics, Health and Therapeutics, University of Leeds, Clarendon Way, Leeds LS2 9JT, UK; 3 Department of Clinical Biochemistry, Leeds Teaching Hospitals NHS Trust, Leeds General Infirmary, Great George Street, LeedsLS1 3EX, UK; 4 Division of Human Development at Nottingham University Hospitals NHS Trust, Department of Obstetrics and Gynaecology, Queen's Medical Centre Campus, Nottingham NG7 2UH, UK

**Keywords:** human granulosa cells, polycystic ovary syndrome, metformin, energy metabolism, progesterone

## Abstract

**STUDY QUESTION:**

What are the consequences of polycystic ovary syndrome (PCOS) pathology and metformin-pretreatment *in vivo* in women with PCOS on the metabolism and steroid production of follicular phenotype- and long-term cultured-granulosa cells (GC)?

**SUMMARY ANSWER:**

PCOS pathology significantly compromised glucose metabolism and the progesterone synthetic capacity of follicular- and long-term cultured-GCs and the metabolic impact of PCOS on GC function was alleviated by metformin-pretreatment *in vivo*.

**WHAT IS KNOWN ALREADY:**

Granulosa cells from women with PCOS have been shown to have an impaired insulin-stimulated glucose uptake and lactate production *in vitro.* However, these results were obtained by placing GCs in unphysiological conditions in culture medium containing high glucose and insulin concentrations. Moreover, existing data on insulin-responsive steroid production *in vitro* by PCOS GCs vary.

**STUDY DESIGN, SIZE AND DURATION:**

Case-control experimental research comparing glucose uptake, pyruvate and lactate production and progesterone production *in vitro* by GCs from three aetiological groups, all undergoing IVF; healthy control women (Control, *n* = 12), women with PCOS treated with metformin *in vivo* (Metformin, *n* = 8) and women with PCOS not exposed to metformin (PCOS, *n* = 8). The study was conducted over a period of 3 years between 2007 and 2010.

**PARTICIPANTS/MATERIALS, SETTING, METHODS:**

Rotterdam criteria were used for the diagnosis of PCOS; all subjects were matched for age, BMI and baseline FSH. Individual patient cultures were undertaken with cells incubated in a validated, physiological, serum-free culture medium containing doses of 0–6 mM glucose and 0–100 ng/ml insulin for 6 h and 144 h to quantify the impact of treatments on acute and long-term metabolism, respectively, and progesterone production. The metabolite content of spent media was measured using spectrophotometric plate reader assay. The progesterone content of spent media was measured by enzyme-linked immunosorbent assay. Viable GC number was quantified after 144 h of culture by the vital dye Neutral Red uptake assay.

**MAIN RESULTS AND THE ROLE OF CHANCE:**

Granulosa cells from women with PCOS pathology revealed reduced pyruvate production and preferential lactate production in addition to their reduced glucose uptake during cultures (*P* < 0.05). Metformin pretreatment alleviated this metabolic lesion (*P* < 0.05) and enhanced cell proliferation *in vitro* (*P* < 0.05), but cells retained a significantly reduced capacity for progesterone synthesis compared with controls (*P* < 0.05).

**LIMITATIONS, REASONS FOR CAUTION:**

Although significant treatment effects were detected in this small cohort, further studies are required to underpin the molecular mechanisms of the effect of metformin on GCs.

**WIDER IMPLICATIONS OF THE FINDINGS:**

The individual patient culture strategy combined with multifactorial experimental design strengthens the biological interpretation of the data. Collectively, these results support the notion that there is an inherent impairment in progesterone biosynthetic capacity of the GCs from women with PCOS. The positive, acute metabolic effect and the negative long-term steroidogenic effect on GCs following metformin exposure *in vivo* may have important implications for follicular development and luteinized GC function when the drug is used in clinical practice.

**STUDY FUNDING/COMPETING INTEREST(S):**

No competing interests. This work was supported by the UK Medical Research Council Grant Reference number G0800250.

## Introduction

Polycystic ovary syndrome (PCOS) is a condition of primary ovulatory dysfunction associated with metabolic disturbances (Wild *et al.*, 2000; Venkatesan *et al.*, 2001; Wijeyaratne *et al.*, 2002; ESHRE/ASRM, 2004). The consensus definition of PCOS by the European Society for Human Reproduction and Embryology, and the American Society for Reproductive Medicine (ESHRE/ASRM) (ESHRE/ASRM, 2004) is used widely in practice and requires the presence of two out of three features: oligo/amenorrhoea, clinical or biochemical features of hyperandrogenism and/or ultrasound feature of polycystic ovaries. A state of insulin resistance, compensatory hyperinsulinaemia and perturbed glucose metabolism of somatic cells may occur in women with PCOS (Book and Dunaif, 1999; Shulman, 2000). Insulin-mediated uptake of glucose by peripheral tissues is reduced by 35–45% in women with PCOS when compared with age and weight matched controls (Dunaif, 1997).

Recently, marked differences were observed in the way energy substrates were metabolized by sheep follicular GCs compared with other species (Campbell *et al.*, 2010). Glucose, metabolized under anoxic conditions to lactate, was shown to be the preferred energy substrate required to support the gonadotrophin-induced differentiation of sheep GCs *in vitro*; fructose and pyruvate, but not galactose, were consumed as alternative sources of energy. In normal human ovaries, it is established that the GCs metabolize glucose by glycolysis and this results in the production of lactate and pyruvate (Billig *et al.*, 1983; Harlow *et al.*, 1987; Boland *et al.*, 1993). While the pyruvate so derived is utilized by the oocytes as their energy source, lactate either diffuses into the blood or is converted back to pyruvate (Leese and Lenton, 1990; Boland *et al.*, 1994). The extent of aberrant metabolism present in the GCs from women with PCOS has not been fully investigated (Willis and Franks, 1995; Rice *et al.*, 2005). Indeed, Purcell *et al.* has demonstrated blunted insulin-stimulated glucose uptake *in vitro* by cumulus cells from women with PCOS (Purcell *et al.*, 2012). Previous researchers have also shown that there is an impaired insulin-stimulated lactate production but intact insulin-responsive steroid production by follicular GCs from women with PCOS (Lin *et al.*, 1997; Fedorcsak *et al.*, 2000; Rice *et al.*, 2005). Previous studies which have cultured GC over 48 h *in vitro* have demonstrated that insulin-induced glucose uptake by GCs in response to increasing doses of insulin is much lower in GCs from anovulatory patients with PCOS than from women with either normal ovaries or ovulatory polycystic ovaries (Rice *et al.*, 2005). However, caution needs to be exercised as many of these results were obtained by placing the cells in highly unphysiological conditions in serum-containing medium with high glucose and insulin concentrations.

Although insulin has long been recognized as the hormone of glucose utilization (Lieberman and Marks, 2009), its role in follicle glucose metabolism and maturation is poorly understood in women with normal ovarian function as well as in women with PCOS. Furthermore, both non-insulin and insulin-dependent glucose transporters (GLUTs), renamed as SLC2A1 and SLC2A4, respectively, have been identified in sheep ovaries (Campbell *et al.*, 2010). Moreover, the expression of SLC2A1 and 4 *in vitro* by sheep GCs was modulated by the dose of glucose in the medium. So there are internal mechanisms other than insulin-mediation likely to be involved in glucose transport by GCs. In insulin-responsive tissues, however, insulin plays a key role in signalling for mobilization of GLUT to the cell membrane, facilitating glucose entry into the cell (Venkatesan *et al.*, 2001). The rate-limiting factor in insulin-mediated glucose uptake is the transfer of GLUTs to the plasma membrane (Girard *et al.*, 1992; Takata, 1996; Scheepers *et al.*, 2004). Tyrosine phosphorylation of the insulin receptor (IR) activates the whole insulin cascade and results in glucose uptake by the cells (Dunaif, 2006). Alternatively, serine phosphorylation of the IR may inhibit the insulin cascade (Book and Dunaif, 1999). Whilst there is inconsistency as to which component of the insulin-mediated glycolytic pathway is affected in GCs in women with PCOS, excessive serine phosphorylation has been attributed to the post-binding defect and insulin resistance observed in these women (Prelevic, 1997; Shulman, 2000; Poretsky *et al.*, 2001; Seto-Young *et al.*, 2003).

Since insulin resistance and hyperinsulinaemia are central to the pathogenesis of PCOS, it is natural to expect insulin sensitisers to be of benefit in the management of the condition (Pasquali and Gambineri, 2009). Metformin, an insulin sensitiser, is the first-line oral antihyperglycemic drug used in the management of type 2 diabetes mellitus. In insulin-treated type 2 diabetic patients, addition of metformin improves insulin sensitivity and glycemic control while allowing a reduction in the daily insulin dose (Cusi and DeFronzo, 1998). Metformin alone decreases fasting plasma glucose concentration to 4–4.5 mM and HbA1c by 1.5–2.0% in patients with type 2 diabetes mellitus. In addition, metformin has been reported to decrease lipid oxidation and plasma free fatty acid levels thereby improving the cardiovascular risk profile in type 2 diabetic patients. In the U.K. Prospective Diabetes Study, treatment with metformin showed a significant reduction in diabetes-related death, heart attacks and stroke (Wright *et al.*, 1998).

The metabolic actions of metformin on cells include increases in insulin sensitivity of responsive tissues, IR number and affinity in skeletal muscle and adipose cells, conversion of glucose to lactate by erythrocytes, tyrosine kinase activity and glucose uptake (Cusi and DeFronzo, 1998; Musi *et al.*, 2002). Conversely, metformin results in a decrease in glucose absorption by gut, plasma glucagon levels, gluconeogenesis and glycogenolysis in the liver (Sarabia *et al.*, 1992; Santos *et al.*, 1997; Cusi and DeFronzo, 1998; Wiernsperger and Bailey, 1999; Musi *et al.*, 2002).

Chemically, metformin belongs to the biguanide group of compounds and is made of *N*,*N*-dimethylimidodicarbonimidic diamide hydrochloride as indicated below.

Metformin has an absolute oral bioavailability of 40–60% within 6 h of ingestion and a half-life of 4.0–8.7 h (Robert *et al.*, 2003). The total daily therapeutic dose of metformin may be 0.5–3 g usually split into 2 or 3 doses (Scheen, 1996). It is not bound to any protein and no metabolites have been identified that circulate in the bloodstream (Scheen, 1996). Side effects of metformin are primarily confined to the gastrointestinal tract such as abdominal discomfort and diarrhoea (Stadtmauer *et al.*, 2001; Tang *et al.*, 2006a,b). Lactic acidosis is rare, with an incidence of three cases per 100 000 patient-years of therapy, mainly in those with impaired renal function (Wiholm and Myrhed, 1993).

A key perturbation in women with PCOS is excessive ovarian androgen production and consequently, excessive estrogen production by GCs. Although the improvement in androgen concentrations in the circulating and follicular environment of women with PCOS treated with metformin may bring indirect beneficial effects to the developing oocytes (Moghetti *et al.*, 2000; Palomba *et al.*, 2010), any direct effects of metformin on GC glucose metabolism have yet to be identified. Recently, porcine GCs cultured *in vitro* for 24 h in the presence of metformin and insulin demonstrated enhanced mRNA expressions of insulin receptor substrate-1 (IRS-1) and insulin-like growth factor-1 receptor (IGF-1R), suggesting that metformin could change the metabolic capacity of ovarian GCs (Lee *et al.*, 2012). In the ovary, earlier reports have also characterized various subunits of AMP-activated protein kinase (AMPK) in GCs, theca cells, oocytes and corpora lutea of cows (Fryer *et al.*, 2002). Metformin has been shown to activate the AMPK cascade, which in turn takes part in glucose transport into the muscle cell (Fryer *et al.*, 2002). Indeed, exposure to metformin has been shown to lead to increased tyrosine kinase activity and 2-deoxy glucose transport in a dose-dependent manner in rat smooth vascular smooth muscle cells suggesting enhanced sensitivity to insulin and IGF-1 (Dominguez *et al.*, 1996).

The objective of this research was therefore to investigate the capacity for glucose uptake and utilization by human GCs in normal women and women with PCOS with or without prior treatment *in vivo* with metformin with a view to understanding the consequences of the PCOS pathology on the metabolism of the energy resources available to the oocyte during its development within the follicle. For this, glucose uptake and pyruvate and lactate production by GCs were quantified over an acute time frame and after long-term culture. Acute measures of GC metabolism and progesterone production 6 h after oocyte retrieval (OR) reflect the functional capacity of follicular phenotype GCs at the time of oocyte harvest. In contrast, metabolic and steroid measurements made after long-term culture reflect the inherent capacity of GC to adapt and respond to defined glucose and insulin environments *in vitro*. Cultures were established from GCs harvested from individual patients to facilitate direct comparison of GC metabolism *in vitro* with plasma follicular fluid metabolites in the same patient.

## Materials and Methods

### Ethical approval

The York Research Ethics Committee approved the research protocol. Reference number: 06/Q1108/76. Written informed consent was obtained from all participants.

### Patients

The study population contained three aetiological subgroups, namely, women with normal ovaries (Control *n* = 12), women with PCOS not treated with metformin during their IVF/ICSI treatment (PCOS *n* = 8) and women with PCOS treated with metformin during their IVF/ICSI treatment (Metformin *n* = 8). Within each aetiological group, the number of subjects completing different components of the GC research varied. Relevant *n* values are given in respective tables and graphs.

Women attending the assisted conception units at Leeds General Infirmary and St James's University Hospital, aged 20–39 years with normal serum FSH concentrations (1–8 IU/l) were recruited to donate their GCs for research. Those women who met the Rotterdam criteria (ESHRE/ASRM, 2004) were classified as having PCOS. Full blood count and liver function tests were conducted in women with PCOS before receiving metformin. Those women with a BMI >30 kg/m^2^ also underwent a glucose tolerance test and fasting lipid profile (Wijeyaratne *et al.*, 2002). Women with impaired glucose tolerance and diabetes were excluded from the study.

Study participants received a long agonist protocol for their IVF treatment. Nafarelin acetate (200 mcg three times per day, Pharmacia, Milton Keynes, UK), buserelin (0.5 mg per day, Pharmacia, Milton Keynes, UK) or leuproline acetate (Prostap 3.75 mg single dose, Wyeth Laboratories, Hants, UK) was used for pituitary down-regulation. Controlled ovarian hyperstimulation was achieved following delivery of either urinary or recombinant FSH. None of the participants received a mixed stimulation protocol. For those women receiving metformin, treatment was commenced at a dose of 850 mg twice daily from the day of start of down-regulation with GnRH agonist and continued until the day of OR. The follicular response was considered to be satisfactory for an adequate yield of GCs when there were at least 10 follicles of at least 13 mm diameter present on the transvaginal ultrasound scan on the last day of stimulation, of which at least 3 follicles were ≥17 mm in diameter. Ovulation was triggered in all subjects from the three groups by injection of 5000–10 000 IU of the same hCG preparation (Pregnyl, Organon, Welwyn Garden City, Herts, UK) and transvaginal oocyte retrieval was performed 34–36 h later. On the day of OR, peripheral blood samples were obtained for the estimation of fasting serum glucose and insulin concentrations. At the time of transvaginal OR, a clean sample of follicular fluid was collected from one or both ovaries. Cellular contaminants were removed by centrifugation at 900 g for 10 min prior to storing the supernatant at −80°C for later analysis. Following recovery of oocytes, the residual GCs in the follicular aspirates from individual patients were processed for culture.

### Granulosa cell harvest and culture

Reagents were purchased from Sigma-Aldrich Company Ltd, (Dorset, UK) unless otherwise stated. Modified McCoy's 5A medium (SAFC Biosciences, Hampshire, UK) without glucose and glutamine was used as the base medium for GC preparation and culture. Medium was supplemented with l-glutamine (210 mg/l), 0.1% human serum albumin (C.A.F-D.C.F. cvba-scrl, Brussels, Belgium), Penicillin-G/ Streptomycin (100 IU/ml/100 µg/ml), HEPES, human transferrin (5 µg/ml), sodium selenite selenium (5 ng/ml), Long R3 IGF-I (100 ng/ml), recombinant FSH (0.5 mU/ml) (Gonal-F: Merck-Serono, UK) and recombinant hCG (0.05 mU/ml) (Ovitrelle: Merck-Serono, UK). All media were pre-equilibrated to 37°C and 5% CO_2_ in air in a humidified incubator.

Follicular aspirates from individual patients were centrifuged at 900 g at room temperature. Blood contaminants were removed from GCs by Histopaque 1700 gradient centrifugation at 900 g at room temperature. The GC pellet was resuspended in 20 ml volume of medium and washed by a further 10 min centrifugation. The GC pellet was resuspended in 1 ml of GC preparation medium containing 0.02% (w/v) EDTA and gentle repeated pipetting was carried out to break up cellular clumps. After further washing, the cell stock was finally resuspended in 1 ml of GC culture medium.

Viable GC number was counted by trypan blue dye exclusion (Picton *et al.*, 1999) and cells were seeded at a concentration of 1 × 10^5^ viable cells per well into 250 µl of McCoy's 5A culture medium which had been further supplemented with varying doses of insulin (0, 0.01, 0.1, 1, 10 and 100 ng/ml) and glucose (0, 3 and 6 mM). Cells were cultured in 96-well flat bottom Nunc culture plates (NUNC, Scientific Laboratory Supplies, Wilford, Nottingham, UK) at 37°C and 5% CO_2_ in a humidified incubator. Each glucose/insulin treatment was tested in triplicate for each patient. In the physiological, serum-free culture conditions used in the present experiments the GCs did not attach to the plastic matrix of the culture dish nor did they form the flattened, fibroblastic monolayers which are typical of serum- and/or extracellular matrix-based culture systems. In the culture conditions reported here, the GCs formed discrete, free-floating, 3-dimensional clumps of rounded cells which were resonant of the follicular phenotype of GCs seen *in vivo*. These non-adherent GC cultures did not attain confluence *in vitro*.

For assessment of acute GC metabolism reflective of the follicular environment, spent culture media was collected after 6 h of incubation by the removal and replacement of 140 µl of medium. Thereafter, culture media was refreshed at 48, 96 and 144 h by removal and replacement of 150, 200 and 150 µl of media, respectively. The 6 h incubation time used to quantify acute, follicular GC metabolism at the time of OR was based on the outcome of a series of 10 replicate, individual patient validation cultures and reflected the minimal time required to accurately quantify the turnover of all three metabolites by 1 × 10^5^ viable GCs *in vitro*. In the long-term culture studies, the intermediate media change time points and total duration of culture (144 h) used have been extensively validated for the same culture system using ovine, porcine and bovine GCs and have been shown to provide accurate time points for the measurement of steroidogenesis and metabolism by GCs in response to defined culture conditions *in vitro* (Campbell *et al.*, 1996; Picton *et al.*, 1999). Spent media were stored at –80°C for assays of glucose, pyruvate and lactate turnover and progesterone production. After 144 h of culture, GC number and viability was evaluated by measuring the uptake of the vital dye, Neutral Red (Campbell *et al.*, 1996; Picton *et al.*, 1999).

The glycolytic index ratio is expected to be 2:1 when glucose is fully metabolized by oxygen-independent glycolysis to its final end product lactate via pyruvate (Harris *et al.*, 2007). A lower ratio is indicative of a drop in the formation of lactate whereas a higher ratio is indicative of lactate production from extra sources such as intracellular glycogen in addition to oxygen-independent glycolysis of the exogenous glucose supplied to the cells.

### Serum and follicular fluid glucose and insulin assays

Serum and follicular fluid glucose and insulin assays were carried out at the routine Clinical Biochemistry Laboratory at Leeds General Infirmary. Serum and follicular fluid glucose was measured by the glucose oxidase method using ADVIA systems (Siemens Healthcare Diagnostics, Inc., Camberley, UK), designed for estimation in human serum, plasma, urine and other body fluids. The analytical range was 0–41.6 mmol/l. All assays were performed according to manufacturer's instructions. Glucose was measured after enzymatic oxidation in the presence of glucose oxidase. Routine Quality Controls (QCs), both internal and external, were performed as per the laboratory quality standards. The between-batch coefficient of variation (CV) was <5%.

Serum insulin concentrations were measured by immunochemiluminometric assay using ADVIA Centaur Insulin (Siemens Healthcare Diagnostics), designed for measurement of insulin in serum and plasma. The results were reported in mU/l and converted to ng/ml for presentation. Insulin resistance was calculated using the quantitative insulin sensitivity check index method (QUICKI = 1/((log (fasting insulin µU/mL) + log (fasting glucose mg/dl))) (Mather *et al.*, 2001).

To validate the measurement of follicular fluid insulin, insulin standards were prepared using recombinant human insulin, 3 IU/vial (National Institute for Biological Standards and Control (NIBSC), 1st International reference preparation 66/304). Insulin recovery from diluted follicular fluid was checked in triplicates and ranged from 81 to 98%. Recovery of exogenous insulin was validated by addition of 10 μl of insulin stock with a known concentration of insulin to 1 mL of follicular fluid followed by serial dilution and the recoveries ranged from 91 to 118%. After validation, the assays were run according to the ADVIA Centaur Reference Manual. All samples from follicular fluid were analysed in a single batch. The within-assay CVs were 3, 5 and 4% at low (18.6–27.9 pmol/l), medium (69.6–86.6 pmol/l) and high (203.1–252.1 pmol/l) concentrations of insulin-QCs, respectively.

### Assays of GC metabolism

Metabolites were measured using enzyme-linked spectrophotometric/fluorometric assays on a 96-well plate reader (Biolinx MRX plate reader, Dynatech Laboratories, San Diego, CA, USA). The concentrations of metabolites measured in the spent media at each time point were used to calculate the rate of metabolism of viable cells per well after 144 h of culture, after accounting for any carry-over of metabolites during media changes at the intermediate time points. The colour changes for each metabolite were proportional to the amount of glucose, pyruvate and lactate in the spent media. Lactate was measured by monitoring the increase in fluorescence due to the conversion of NAD to NADH; pyruvate by a decrease in fluorescence due to the oxidation of NADH; and glucose by an increase in fluorescence due to the formation of NADPH from NADP (Hugentobler *et al.*, 2008). The assay sensitivity was 0–10 mM for glucose/lactate and 0–2 mM for pyruvate. The assay reaction time was 10, 30 and 5 min for glucose, lactate and pyruvate, respectively. Each metabolism assay included cell-free culture controls and assay control QCs spiked with known concentrations of metabolites (low and high concentration within the sensitivity range). The intra- and inter-assay CVs were <10% for all metabolites except for the acute culture pyruvate assays where the CV was <20%.

### Progesterone assays

Progesterone measurements were carried out using an enzyme-linked immunosorbent assay in the laboratory at the Division of Obstetrics and Gynaecology, School of Clinical Sciences, Queens Medical Centre, Nottingham University. Assays were performed utilizing a progesterone antibody (SAPU R7044X) previously validated for radioimmunoassay (Campbell *et al.*, 1998) in combination with a Progesterone-3-Horse Radish Peroxidase conjugate obtained from Abiox plc (Newberg OR, USA). The limit of detection was <39 pg/ml, and the intra- and inter-assay CVs were 5 and 11%, respectively. The progesterone production rate per cell per hour was calculated from the concentrations of progesterone measured after correction for carry over effects during media changes.

### Statistical analysis

All metabolite and hormone production/consumption data were expressed per hour per 1 × 10^5^ viable GCs. Categorical data in the patient demographics were converted to percentages before analysis. The Chi-squared test was used to investigate differences between categorical variables such as fertilization rate and pregnancy rate. Metabolite and steroid data from all three groups were normalized by log-transformation (log_10_ (*x* + 1)). Viable cell number data after 144 h of culture were log transformed prior to analysis. A multivariate analysis of variance (ANOVA) was used to test the effects of culture treatment on cell metabolism; Bonferroni *post hoc* test was used to test the difference between means. Univariate-repeated measure ANOVA was carried out to determine the effects of insulin and glucose concentrations on cultured GC between 6 h and 144 h of culture. In all cases, *P* < 0.05 was considered to be statistically significant. Both, STATA 10.2 and SYSTAT 15, statistical packages were used appropriately for data analysis. The glycolytic index was calculated as the ratio of number of moles of lactate produced from one mole of glucose consumed.

## Results

### Patient demographics

The subjects from all three groups were matched for their age, duration of infertility, BMI and baseline serum FSH (Table I). All groups of patients received a comparable total dose of FSH over a similar duration for controlled ovarian hyperstimulation. The number of mature oocytes per patient was lower in the PCOS group than the Controls and the difference just reached significance (*P* = 0.046). The fertilization rate per metaphase II oocyte, positive pregnancy rate and clinical pregnancy rates were similar in all three groups.

**Table I:** Demographics of the participants of the granulosa cell metabolism studies.

Clinical data	Control (*n* = 12)	PCOS (*n* = 8)	Metformin (*n* = 8)	Significance
Age (years)	34.4 ± 1.4	31.5 ± 1.2	34.4 ± 1.3	ns
Duration of infertility (years)	3.2 ± 0.7	4.1 ± 0.8	3.4 ± 0.7	ns
BMI (kg/m^2^)	25.5 ± 1.6	26.0 ± 1.9	29.8 ± 1.5	ns
Serum FSH (IU/l)	6.8 ± 0.6	5.5 ± 0.5	5.7 ± 0.5	ns
Total days of stimulation	10.2 ± 0.2	12.4 ± 0.9	10.4 ± 0.5	ns
Total dose of exogenous FSH IU	2405 ± 264	2692 ± 382	1885 ± 303	ns
Total number of follicles developed	23.3 ± 2.0	31.1 ± 3.0	23.8 ± 4.0	ns
Number of MII oocytes/patient	16.3 ± 2.0	12.4 ± 2.8	11.1 ± 1.0	PCOS versus Control, *P* = 0.046 Others – ns
Fertilization rate/MII oocyte	67 ± 0.2%	67 ± 0.2%	60 ± 0.2%	ns
Positive pregnancy rate per cycle started	50%	75%	63%	ns
Clinical pregnancy rate per cycle started	50%	57%	44%	ns

The values are mean ± SEM except for fertilization, positive pregnancy and clinical pregnancy rate. Chi^2^ and Kruskal–Wallis tests were used for statistical analysis.

PCOS: polycystic ovary syndrome; MII: metaphase II; Metformin: women with PCOS who had been treated with metformin.

There were no differences in the mean fasting serum concentrations of glucose, insulin or insulin resistance index between the three treatment groups (Table II). Similarly, there were no significant differences in the mean follicular fluid glucose and insulin concentrations between the three groups and there was no statistically significant relationship between serum and follicular fluid glucose and insulin.

**Table II:** Comparison of biochemical parameters between three groups.

	Sample type	Control	PCOS	Metformin	Significance
Fasting glucose (mM)	Serum	4.5 ± 0.1 (*n* = 9)	4.2 ± 0.1 (*n* = 8)	4.5 ± 0.3 (*n* = 8)	ns
Fasting insulin (ng/ml)	Serum	0.2 ± 0.1 (*n* = 9)	0.4 ± 0.1 (*n* = 8)	0.3 ± 0.1 (*n* = 8)	ns
QUICKI insulin resistance index	Serum	0.41 ± 0.1 (*n* = 9)	0.4 ± 0.3 (*n* = 8)	0.4 ± 0.2 (*n* = 8)	ns
Glucose (mM)	Follicular fluid	2.7 ± 0.9 (*n* = 4)	3.2 ± 0.2 (*n* = 5)	3.3 ± 0.5 (*n* = 6)	ns
Insulin (ng/ml)	Follicular fluid	50 ± 21 (*n* = 4)	14 ± 10 (*n* = 5)	41 ± 9 (*n* = 6)	ns

The values are mean ± SEM for the number of analyses shown. Comparison is between the three aetiological groups for each biochemical parameter using Kruskal–Wallis test. Samples of clean catch follicular fluid were not available for all patients who consented for GC metabolism research. Therefore fewer follicular fluid samples were available for glucose and insulin measurements.

### Acute GC metabolism

#### Effect of aetiology on acute granulosa cell metabolism

**Figure 1:**
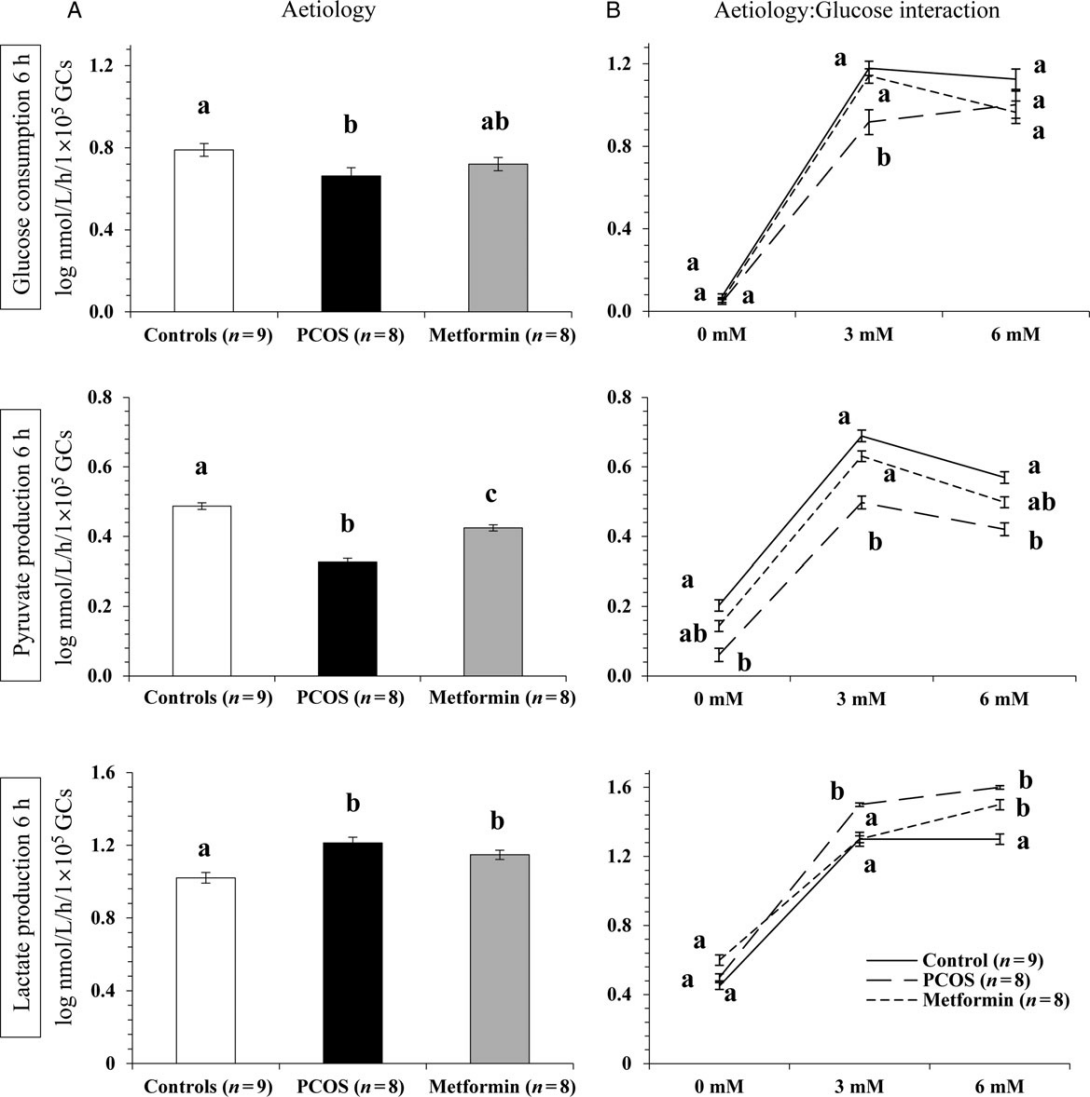
The effect of aetiology (**A**) and aetiology-glucose interaction (**B**) on acute glucose consumption, pyruvate and lactate production by granulosa cells (GCs) at 6 h of culture. Values plotted are mean ± SEM of the log-transformed data for the number of individual patient cultures shown. Data have been standardized according to viable cell number plated. Different letters on graphs in (A) indicate significant differences between the three aetiological groups tested for each metabolite (*P* < 0.05). Different letters on graphs in (B) indicate significant differences (*P* < 0.05) between the three aetiological groups within each glucose dose.

There was no demonstrable effect of insulin concentration in the culture medium on acute glucose consumption, or pyruvate and lactate production, justifying pooling of all results for various insulin doses. There was, however, a significant effect of patient aetiology on acute (6 h) GC glucose consumption, and pyruvate and lactate production (*P* < 0.001, Fig. 1A). Overall, glucose consumption and pyruvate production by GCs from women with PCOS were significantly (*P* < 0.05) lower than that of Controls. Conversely, GCs from women with PCOS not exposed to metformin *in vivo* produced significantly (*P* < 0.05) more lactate than Control GCs. Metformin-pretreatment *in vivo* appeared to ‘normalize’ the glycolytic pathway of GCs by increasing their acute pyruvate production and decreasing their mean lactate production. However, lactate production by metformin-pretreated GCs still remained significantly (*P* < 0.05) higher than that of Control GCs (Fig. 1A).

#### Effect of glucose concentration on acute GC metabolism

There was a significant (*P* < 0.05) effect of the glucose concentration in the culture medium on acute glucose consumption, pyruvate and lactate production by human GCs *in vitro*. As expected, glucose consumption was negligible in cells cultured in glucose-free media but there was no difference in the glucose uptake between cells incubated in 3 and 6 mM glucose. Cells lacking glucose produced little pyruvate *in vitro* but when glucose was available in the medium, there was a significant (*P* < 0.001) dose-responsive decrease in overall pyruvate production; 3.5 ± 0.1 nmol/l/h/1 × 10^5^ viable GCs at 3 mM glucose compared with 2.5 ± 0.1 nmol/l/h/1 × 10^5^ viable GCs at 6 mM glucose in the medium. Lactate production was limited (2.8 ± 0.2 nmol/l/h/1 × 10^5^ viable GCs) in the absence of glucose, possibly from conversion of the intracellular glycogen reserve. There was an overall significant (*P* < 0.001) rise in lactate production, which corresponded to the increase in glucose in the medium (31.1 ± 1.1 nmol/l/h/1 × 10^5^ viable GCs for 3 mM glucose and 39.5 ± 1.5 nmol/l/h/1 × 10^5^ viable GCs for 6 mM glucose).

#### Interaction between aetiology and glucose concentration on acute GC metabolism

There was a significant (*P* < 0.001) interaction between patient aetiology and the glucose concentration in the GC incubation medium that influenced acute GC metabolism *in vitro* (Fig. 1B). At the 3 mM physiological glucose concentration, exposure of PCOS GCs to metformin *in vivo* significantly (*P* < 0.05) increased acute glucose uptake and pyruvate production, bringing the turnover of these two metabolites closer to the Control group. Glucose consumption by PCOS GCs was significantly (*P* < 0.05) lower than Control and Metformin GCs when incubated with 3 mM glucose (Fig. 1B).

There was no significant difference in glucose consumption between the three groups when the media glucose concentration was 6 mM. While pyruvate production by Control GCs was significantly (*P* < 0.001) higher than that by the PCOS GCs at 6 mM glucose, metformin-exposed GCs were mid-way between the PCOS and Control groups at 6 mM glucose dose (Fig. 1B).

Granulosa cells from women with PCOS produced significantly (*P* < 0.05) more lactate than Control GCs at both 3 and 6 mM glucose. Furthermore, GC lactate production on incubation with 3 mM glucose *in vitro* was significantly (*P* < 0.001) reduced relative to PCOS cells following metformin-pretreatment *in vivo* matching the levels recorded for Control cells (Fig. 1B).

### Effect of metformin exposure *in vivo* on viable GC number *in vitro*

**Figure 2:**
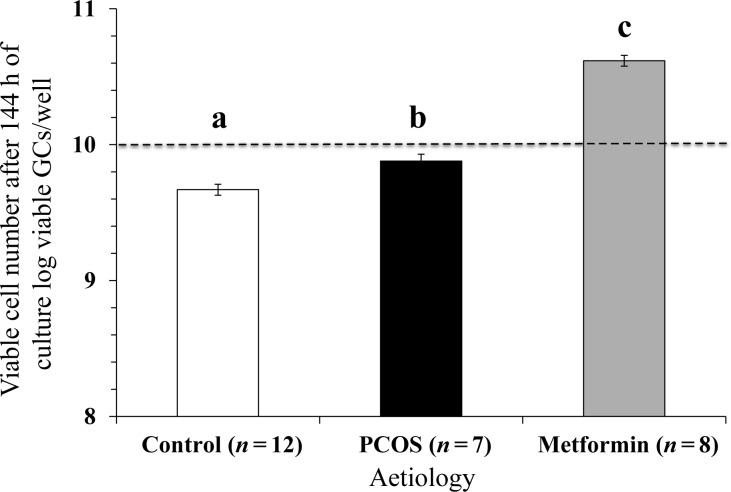
The effect of aetiology on GC viability after 144 h of culture. The values plotted are mean ± SEM of the log-transformed data for the number of individual patient cultures shown. Different letters on the graph indicate significant differences (*P* < 0.05) between the aetiologies. The dotted line represents the plating density of viable cells per well at the start of the culture.

The GC viability was quantified in all those subjects whose GCs successfully completed 144 h of culture. The results showed a significant change in the number of viable cells between the start and the end of extended culture (Fig. 2). There was no effect of glucose or insulin dose including zero doses on viable GC number at the end of the extended cultures; hence the results were pooled for all glucose and insulin doses. There was, however, a strong effect of aetiology on GC viability such that, exposure to metformin *in vivo* resulted in a significantly higher number of viable GCs (*P* < 0.05, Fig. 2) after 144 h of culture compared with GCs from the PCOS or Control groups.

### Long-term GC metabolism

Glucose, pyruvate and lactate metabolism remained detectable in GCs after 144 h of culture. There was no demonstrable effect of insulin at any of the doses tested (data not shown), justifying pooling of data. Glucose consumption and utilization by GCs was detected in the absence of insulin supplementation. There was also no significant interaction between aetiology and insulin content in the medium following long-term culture of GCs.

#### Effect of aetiology on long-term GC metabolism

**Figure 3:**
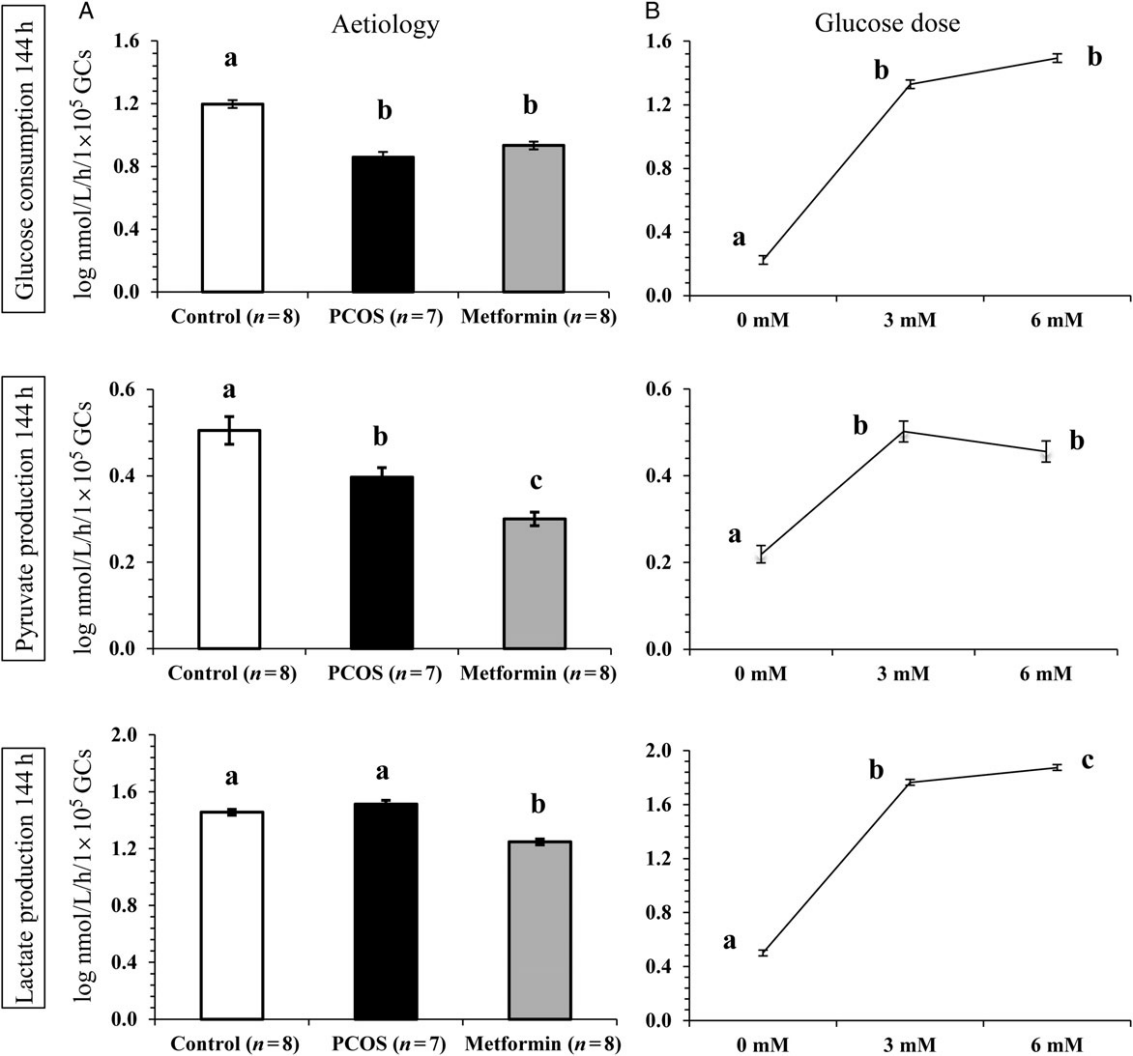
The effect of aetiology (**A**) and glucose dose in the culture medium (**B**) on glucose consumption, pyruvate and lactate production by GCs after 144 h of culture. The values are mean ± SEM of the log-transformed data for the number of individual patient cultures shown. Data have been standardized according to viable cell number measured after 144 h of culture. Different letters on the graphs indicate significant differences (*P* < 0.05) between the three aetiological groups and the glucose doses tested for each metabolite.

Overall glucose uptake by Control GCs was significantly (*P* < 0.05) higher than that of PCOS and PCOS GCs from women pretreated with metformin *in vivo* prior to GC recovery. Control GCs metabolized significantly (*P* < 0.0001) more glucose than PCOS and Metformin GCs (Fig. 3A). There was a clear effect of aetiology on pyruvate production such that the Control GCs produced significantly (*P* < 0.0001) more pyruvate than the other two groups at all doses of glucose and insulin tested. Pyruvate production by PCOS GCs was less than that of Control GCs, but higher than Metformin GCs (*P* < 0.01 and *P* < 0.05, respectively, Fig. 3A). Mean lactate production by GCs for the three aetiologies is also shown in Fig. 3A. Although the PCOS GCs produced more lactate than Control GCs, this difference was not significant for either 3 mM or 6 mM glucose. The metformin-pretreated GCs produced significantly (*P* < 0.0001) less lactate than either Control GCs or their PCOS counterparts for all glucose doses tested (Fig. 3A).

#### Effect of glucose concentration on long-term GC metabolism

The glucose concentration in the medium significantly (*P* < 0.0001) influenced the overall glucose uptake by the GCs but the observed difference was mainly between GCs cultured in the absence versus the presence of glucose in the medium rather than between 3 and 6 mM glucose doses (Fig. 3B). Although there was a dose-dependent drop in pyruvate production by all three aetiologies when the glucose supply in the medium was increased from 3 to 6 mM; this effect was not significant (*P* > 0.05). Similar to acute GC metabolism, there was a trend (*P* = 0.053) for an increase in overall lactate production in response to the presence of higher glucose in the medium (Fig. 3B).

#### Interaction between aetiology and glucose dose on long-term GC metabolism

The only metabolite turnover which demonstrated a strong tendency (*P* = 0.05) for an interaction between patient aetiology and glucose dose was lactate production after 144 h of culture. There was a significant (*P* < 0.05) increase in the overall lactate production rate in response to the glucose concentration in the medium in the Control and PCOS groups while the increment was not significant in the Metformin group.

#### Metabolite turnover rate over 144 h

**Figure 4:**
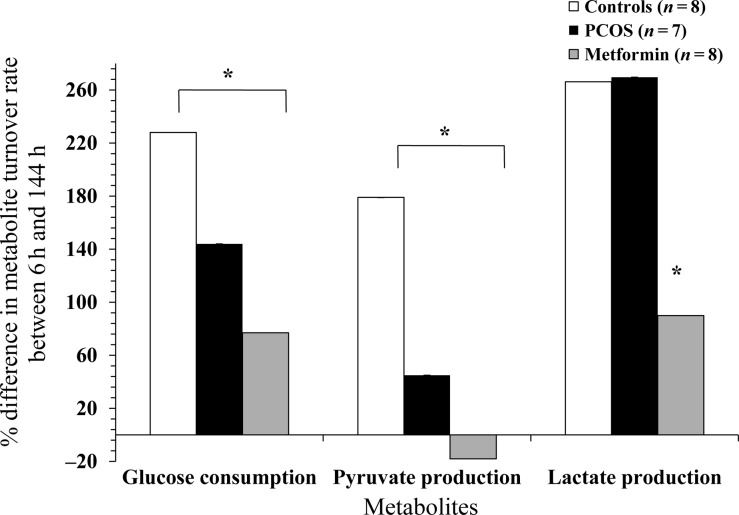
The effects of aetiology on percentage increase (positive values) or decrease (negative values) in glucose consumption and pyruvate and lactate production rate per 1 × 10^5^ viable GCs per well over 144 h of culture. The values plotted are mean ± SEM of the data for the number of individual patient cultures shown. Asterisk indicates significant difference (*P* < 0.05) between the aetiologies for the metabolite shown.

In order to capture the impact of the defined culture conditions on GC metabolism, the mean percentage differences in the rate of GC metabolism per viable cells between 6 and 144 h of cultures were analysed for all three aetiological groups. The results demonstrate significant differences between the aetiologies in the way the GCs utilized glucose and produced lactate and pyruvate over 144 h (Fig. 4).

#### Progesterone production by GCs

**Figure 5:**
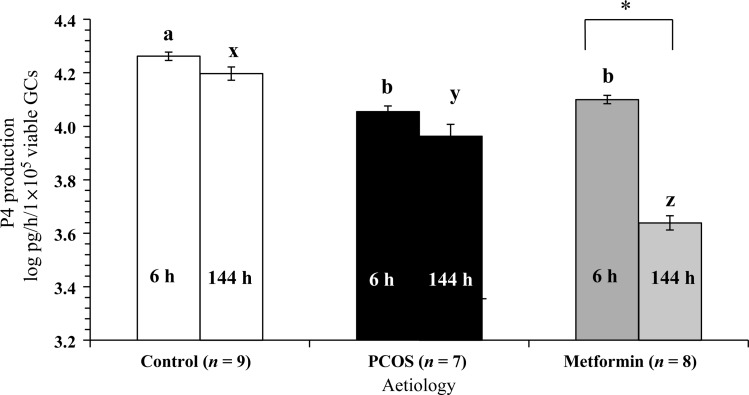
The effects of aetiology and duration of culture on overall progesterone production by GCs. The values plotted are mean ± SEM of the log-transformed data for the number of individual patient cultures shown. Data have been standardized according to viable cell number. a and b indicate significant difference (*P* < 0.05) between the aetiological groups at 6 h of culture (6 h) and x, y and z indicate significant difference (*P* < 0.05) between the aetiological groups at 144 h of culture (144 h). Asterisk indicates significant difference (*P* < 0.05) within aetiology at the two time points shown.

Progesterone production data were available for 24 patients. There was no effect of glucose or insulin doses including zero doses on acute or long-term progesterone production; therefore the data were pooled for further analysis. Measurable progesterone production was detectable for GCs after 6 h of culture and a significant (*P* < 0.0001) difference was observed between the three aetiologies such that Control GCs produced significantly (*P* < 0.0001) more progesterone than either PCOS GCs or metformin-pretreated GCs (Fig. 5). Similarly, after 144 h of culture, there was a significant difference (*P* < 0.0001) in the long-term progesterone production by GCs from the three aetiological groups (Fig. 5). After long-term culture, the highest progesterone synthesis was reported for Control GCs, followed by PCOS GCs and finally by the metformin-pretreated group. Metformin exposure *in vivo* had no significant effect on acute progesterone production compared with untreated PCOS GCs. While the Control and PCOS GCs not exposed to metformin *in vivo* maintained their progesterone production over time, the Metformin GCs significantly (*P* < 0.0001) dropped their progesterone production over 144 h of culture *in vitro*.

#### The glycolytic index of GCs

**Figure 6:**
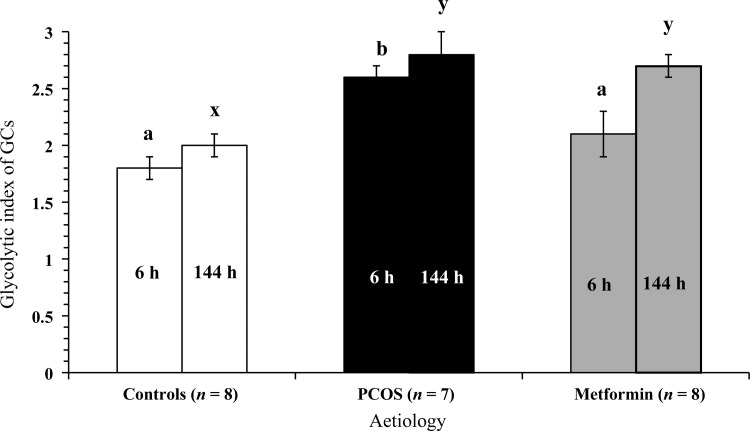
The effects of aetiology and duration of culture on the glycolytic index of GCs. The values plotted are mean ± SEM of the data for the number of individual patient cultures shown. a and b indicate significant difference (*P* < 0.05) between the aetiological groups at 6 h of culture (6 h) and x and y indicate significant difference (*P* < 0.05) between the aetiological groups at 144 h of culture (144 h).

Both aetiology and glucose content of the culture medium had a significant (*P* < 0.001) effect on the acute glycolytic index of GCs. The PCOS GCs demonstrated the highest glycolytic index (2.6 ± 0.2, *n* = 8), relative to metformin-pretreated GCs (2.1 ± 0.1, *n* = 8) and Control GCs (1.8 ± 0.1, *n* = 9) (Fig. 6). The differences in the glycolytic index between Control and PCOS GCs and metformin-pretreated PCOS GCs were significant (*P* < 0.001; *P* < 0.05, respectively). Metformin-pretreatment shifted the glycolytic index of GCs towards that of Control GCs so that there was no statistical difference between the two groups. The acute glycolytic index increased significantly (*P* < 0.01) with increasing glucose concentration in the medium irrespective of patient aetiology and insulin dose (1.8 ± 0.1, *n* = 25 and 2.3 ± 0.1, *n* = 25 at 3 mM and 6 mM glucose supply, respectively). A rise in the glycolytic index in response to the glucose content in the medium is consistent with increased lactate and decreased pyruvate production by the GCs in response to the glucose supply.

In the long-term cultures, once again there was a strong and significant (*P* < 0.0001) effect of patient aetiology on the glycolytic index of GCs. The glycolytic index was lowest in Control GCs (2.0 ± 0.1, *n* = 6) and was significantly (*P* < 0.0001) different to that of PCOS (2.8 ± 0.2, *n* = 7) and Metformin GCs (2.7 ± 0.1, *n* = 8). Unlike after acute (6 h) culture, none of the GCs showed noticeable changes in the glycolytic index in response to glucose content in the medium after extended culture (144 h). Moreover, metformin-pretreatment did not produce any change in the glycolytic index after extended culture. Overall, the metabolism results indicated preferential lactate production by PCOS GCs compared with Control GCs (Fig. 6).

## Discussion

This research has, for the first time, provided detailed insights into glucose metabolism in defined environments *in vivo* and *in vitro* by GCs from women with PCOS with a view to characterizing the exact metabolic lesion in these cells. The results have shown that all three components of glycolysis namely, glucose uptake and pyruvate and lactate production, are perturbed in women with PCOS. Previous studies have indicated that insulin-induced lactate production *in vitro* by GCs was impaired in women with anovulatory PCOS compared with controls (Lin *et al.*, 1997; Fedorcsak *et al.*, 2000; Rice *et al.*, 2005). In contrast, the acute metabolism results from the current study revealed higher lactate production by PCOS GCs than Controls irrespective of the insulin concentration in the culture medium. Similarly, long-term lactate production was also comparable between Control and PCOS GCs without any demonstrable insulin effect. It is to be noted that the previously reported impaired insulin-stimulated lactate production was only recorded after exposure of cells to supra-physiological doses of insulin, which were higher than the top dose (100 ng/ml) used in the present studies (Lin *et al.*, 1997; Fedorcsak *et al.*, 2000; Rice *et al.*, 2005). Caution needs to be exercised in making a direct comparison between published studies and the present work. In the current study, GC metabolism was measured after 6 and 144 h of culture whereas in previous studies, the metabolites were measured after 48–72 h of culture only. Moreover, the current data on GC metabolism were generated on GCs harvested from individual patients cultured in a serum-free culture system containing sub- to supra- physiological doses of glucose and insulin, and physiological concentrations of hCG (0.05 mU/ml) and FSH (0.5 mU/ml) (Picton *et al.*, 1999). In contrast, Rice *et al.* (2005) used an LH dose range of 0, 2.5 and 5 ng/ml (equivalent to 0, 0.01 mU/ml and 0.22 mU/ml of LH) and a range of insulin concentrations up to a higher maximum dose of 1000 ng/ml to stimulate the GC metabolism in a serum-based culture system containing 5.5 mM glucose.

The present study also reports that the glycolytic index of Control GCs was <2, suggesting that each mole of glucose was not fully metabolized to lactate, the end product of oxygen-independent glycolysis, instead metabolized to other molecules like pyruvate (Harris *et al.*, 2009). The index was >2 for PCOS GCs, which is indicative of complete glycolysis of each mole of glucose to two moles of lactate and additional lactate production possibly from intracellular glycogen. The results lead us to hypothesize that preferential conversion of glucose to the intermediate metabolite pyruvate occurs in Control GCs, while the preferential accumulation of the end product of glycolysis lactate occurs in women with PCOS. These data are relevant to women undergoing assisted conception, as pyruvate is the key energy substrate for the pentose phosphate pathway and so plays a central role in generating the nucleotides which are essential for oocyte nuclear maturation and developmental competence (Sutton-McDowall *et al.*, 2010). Lack of pyruvate production by PCOS GCs may, therefore, compromise the developmental capacity and hence the quality of the oocytes from women with this pathology.

An important finding of the current experiments was the lack of demonstrable effects of insulin in the culture medium on acute or long-term glucose metabolism by the human GCs. This observation suggests that insulin resistance is predominantly an *in vivo* phenomenon, which is not consistently present in cultured granulosa lutein cells collected from women with PCOS undergoing assisted conception. In support of this explanation, insulin resistance has not always been demonstrated *in vitro* in somatic cells from women with PCOS. For example, cultures of skeletal muscle cells failed to show differences in insulin sensitivity between PCOS and control subjects (Corbould *et al.*, 2005). Similarly, insulin binding and insulin-responsive basal glucose transport were comparable between control and PCOS adipocytes whereas the insulin sensitivity, as assessed by adenosine-depleted glucose transport activity, was impaired in PCOS adipocytes (Ciaraldi *et al.*, 1997). The possible explanation given for failure to demonstrate insulin resistance *in vitro* in these previous studies was that the factors such as hyperinsulinaemia, hyperandrogenism and hyperglycaemia, contributing to insulin resistance *in vivo* were not operating *in vitro*. Moreover, insulin resistance *in vivo* is believed to be due to post-receptor signalling defects in tyrosine phosphorylation rather than a problem in the IR affinity and function (Kausch *et al.*, 2001; Venkatesan *et al.*, 2001; Dunaif, 2006). Alternatively, factors such as IGF-1 and gonadotrophins may play a more important role than insulin in regulating glucose uptake and utilization by human GCs at least in the present serum-free cultures *in vitro*. In this context, the inclusion of LR3 IGF-1 in the culture medium might have overridden any effect of insulin on cellular glucose metabolism, albeit, such an overriding effect might have required a much higher dose than the physiological level (100 ng/ml) used in the current experiment. Indeed, most of the published reports on glucose metabolism by PCOS GCs have tested the insulin-responsiveness of the cells in the absence of IGF-1 (Lin *et al.*, 1997; Fedorcsak *et al.*, 2000; Rice *et al.*, 2005). When Wu *et al.* (2003) compared the effects of insulin and IGF-1 on human GC glucose metabolism, the results showed that the affinity and effect of IGF-1 was much more potent than that of insulin on GC glucose uptake in women with PCOS. Conversely, some researchers have proposed that the GC response to insulin in women with PCOS is mediated by the IR rather than IGF (Willis and Franks, 1995). Nevertheless, there is increasing support for the notion that a cross-link between the IR and IGF-1R could lead to an overlap of metabolic activities in cells (Binoux, 1995). Moreover, it has been proposed that IR/IGF-1R-hybrid receptors may be present in GCs, leading to uptake of glucose via IGF-1 stimulation (Lopaczynski, 1999).

The mean serum and follicular fluid glucose concentrations (4.4 ± 0.1 mM and 3.1 ± 0.3 mM, respectively) were comparable to that reported by earlier studies (Leese and Lenton, 1990; Phy *et al.*, 2004; Rice *et al.*, 2005; Nafiye *et al.*, 2010). As reported previously in the literature, the follicular fluid (a filtrate of plasma) glucose concentration was much lower than that in serum (Leese and Lenton, 1990) while the opposite was observed for insulin. This observation supports the notion that follicle cells may not contain insulin-sensitive glucose transporters, leading to accumulation of insulin in the follicular fluid (Campbell *et al.*, 2010).

Treatment of women with PCOS with metformin during their IVF cycles clearly ‘normalized’ the acute glucose metabolism in GCs incubated with a physiological concentration of glucose (3 mM). It is likely that the mechanism by which metformin enhances glucose consumption by the GCs from women with PCOS may directly enhance pyruvate production by the same cells. The present findings for GCs are consistent with the metabolic behaviour of metformin-treated hepatocytes in culture that show an increase in insulin-stimulated glucose uptake, IR phosphorylation-activated glucose intake and glycolysis (Fulgencio *et al.*, 2001; Gunton *et al.*, 2003). Enhanced activity of hexokinase and pyruvate kinase was proposed to be responsible for such effects of metformin. Similarly, metformin treatment over a period of 10 weeks increased the glucose uptake by skeletal muscle cells in subjects with type 2 diabetes mellitus via the AMPK pathway (Musi *et al.*, 2002). However, pyruvate production observed in the GCs in the present study was not fully corrected to the Control levels following exposure to metformin *in vivo*. Whether increasing the clinical dose of metformin would further improve GC metabolism is a question for further research. Furthermore, the question of whether the observed metformin-induced improvement in acute GC metabolism is translated into clinical effectiveness needs further investigation. Previously, metformin pretreatment *in vivo* has shown no significant effect on *in vitro* maturation potential of immature sibling PCOS oocytes produced during IVF treatment (Harris *et al.*, 2010). In line with the available literature, the current study showed no difference in the fertilization rate per mature oocyte, positive pregnancy rate per cycle or clinical pregnancy rate per cycle between the groups. A recent meta-analysis by Palomba *et al.* (2013) also confirmed that the use of metformin during IVF/ICSI treatment did not improve pregnancy or live birth rates. In contrast, metformin treatment has been shown to double live birth rates, most probably by reducing the cancellation rate, when the drug is administered during gonadotrophin-stimulated ovulation induction (Palomba *et al.*, 2014).

The underlying mechanism for the observed increase in viable cell number in PCOS and metformin-pretreated GCs is unclear. The GCs from early antral follicles (0.5–2 mm in diameter) have the potential to proliferate in physiological, serum-free culture when stimulated appropriately, while cells from larger antral follicles (>5–6 mm in diameter) more commonly tend to differentiate if viable cell number is maintained *in vitro*, as evidence by their increased capacity to aromatize androgens to estrogens (Breckwoldt *et al.*, 1996; Campbell *et al.*, 1996; Havelock *et al.*, 2004). The granulosa lutein cells used in the present experiments were predominantly harvested from larger antral follicles and, further, they were already terminally differentiated on collection following exposure to the hCG trigger *in vivo*. Any direct impact of exogenous LH on terminal follicular development prior to hCG-induced luteinization was not measured and was perhaps a limitation of the study. The inherently reduced apoptosis of PCOS GCs may contribute to the increased viable cell number observed at the end of long-term cultures. In support of this idea, women with PCOS showed higher rates of proliferation and lower rates of apoptosis in GCs collected from unstimulated follicles (4–8 mm diameter) when compared with normal controls (Das *et al.*, 2008). While activated caspase-3, an enzyme required for cell apoptosis, has been found to be significantly lower, the expression of cell survival proteins, such as islet-activating protein and Bcl gene-2 protein, has been found to be higher in GCs from women with PCOS compared with healthy women. A recent report also suggests that metformin-pretreatment during IVF may directly sensitize human GCs and so result in the increased proliferation of primary GC lines in culture as well as the increased basal cell viability of human granulosa lutein cells *in vitro*, in the absence of any stimulatory effect from insulin or IGF-1 in the media (Sonntag *et al.*, 2005). Indeed, increased viable cell numbers may also be due to an effect of metformin on the AKT pathway (Hennessy *et al.*, 2005) or a direct suppression of cell cycle arrest by metformin that is independent of insulin (Jalving *et al.*, 2010).

It is striking that the PCOS GCs studied in our experimental series consistently produced less progesterone than Control GCs in both the acute and extended cultures, suggesting that there is an inherently reduced capacity to synthesize progesterone in this aetiology. In support of this idea, Doldi *et al.* (1998) has reported reduced progesterone synthesis *in vitro* by luteinized PCOS GCs. Further, FSH and/or IGF-1-induced progesterone secretion in cultures of GCs from 8- to 10-mm-sized unluteinized follicles from women with PCOS was significantly lower than control GCs, supporting inherent perturbation in the steroidogenic capacity of PCOS GCs (Erickson *et al.*, 1992). An important manifestation of the observed reduced progesterone biosynthesis by PCOS GCs *in vivo* may be a luteal phase deficiency of progesterone synthesis and associated poor pregnancy outcomes (Meenakumari *et al.*, 2004; Unfer *et al.*, 2005). Indeed, measurement of luteal phase urinary pregnanediol:creatinine ratio as an index of progesterone secretion has indicated that women with PCOS secret significantly lower levels of progesterone in the early luteal phase (Joseph-Horne *et al.*, 2002) despite a wide variation in the progesterone secretion index. A significantly reduced serum progesterone index was also observed in the early luteal phase of naturally ovulating oligomenorrheic women with PCOS compared with normal fertile women (Fleming *et al.*, 1995). Collectively these results support the notion that there is an inherent impairment in progesterone biosynthetic capacity of the GCs from women with PCOS.

Interestingly, this report has shown that progesterone production by GCs from women with PCOS was not acutely altered by prior exposure to metformin *in vivo*. Moreover, after 144 h of culture both progesterone synthesis and conversion of glucose to pyruvate and lactate by metformin-pretreated GCs were lower than their PCOS counterparts and Control GCs, after data were corrected for viable cell number. These results suggest that metformin exposure *in vivo* not only increased cell proliferation *in vitro* but that it also permanently slowed down the metabolism and progesterone synthetic capacity the GCs *in vitro*. It is unclear whether this negative metabolic effect was due to: reduced endothelial function in densely populated, viable GCs; substrate limitation *in vitro*; or to a direct effect of metformin on cells. These parameters may not be mutually exclusive. A significant reduction in both insulin and IGF-1-stimulated progesterone production *in vitro* has been reported in two previous studies where control and PCOS GCs were incubated with metformin in the culture medium (Mansfield *et al.*, 2003; Tosca *et al.*, 2007). Additionally, human GC expression of *IGF-1* was lower after treatment with metformin prior to IVF compared with control women with PCOS (Santana *et al.*, 2007). A sustained inhibitory effect of metformin on theca cell androgen production was also recorded during *in vitro* exposure to metformin (Attia *et al.*, 2001). Furthermore, metformin appears to exert an insulin-independent suppressive effect on CYP17-lyase and androstenedione production by human ovarian theca-like tumour cells (Attia *et al.*, 2001). Metformin, when added to the culture media, has also been shown to decrease the levels of the expression of steroid acute regulatory protein (StAR) in rat GCs (Tosca *et al.*, 2006). While an assessment of StAR protein levels by western blot analysis of GCs from the current experimental series would have been informative with respect to the mechanism of action of metformin, analysis of this nature was not possible in the current work due to: (i) limited number of GCs available for analysis when using an individual patient culture strategy; and (ii) the need to accurately quantify GC metabolism and steroidogenesis in relation to viable cell number, as assessed by neutral red assay, at the end of culture. Further research is required to investigate the molecular mechanisms of action of metformin on GCs.

In clinical studies, a significant reduction in serum IGF-1 and progesterone concentrations has been reported in women with PCOS treated with metformin at a dose of 850 mg three times a day for a period of 16 weeks (Berker *et al.*, 2004). Conversely, an observational study reported a significant increase in progesterone secretion on continued use of metformin (serum level of 16.97 ng/ml) during the luteal phase as opposed to a lower serum progesterone concentration in women with PCOS not treated with metformin (4.9 ng/ml) (Meenakumari *et al.*, 2004). It has been suggested that metformin may exert a direct inhibitory effect on 3-hydroxy steroid dehydrogenase, StAR and aromatase enzyme activities thereby reducing steroid production by human GCs (Nestler and Jakubowicz, 1997; la Marca *et al.*, 2002; Mansfield *et al.*, 2003). Finally, AMPK activation by metformin is a mechanism favoured by Tosca *et al.* (2007) to account for reduced synthesis of steroids by rat GCs. The clinical implications of the ‘metformin effect’ on human GCs observed in the current study need further clinical research.

## Conclusion

In conclusion, this study demonstrated that regardless of patient aetiology, insulin doses of 0.01–100 ng/ml had no effect on acute or long-term GC metabolism or on progesterone production by GCs cultured in physiological, serum-free conditions. In contrast, supplementation of media with 3–6 mM glucose consistently affected glucose consumption as well as pyruvate and lactate production by follicular GCs and long-term cultured GCs. The PCOS aetiology compromised the capacity of follicular and cultured GC to consume glucose *in vitro*, and this metabolic effect was alleviated by pretreatment with metformin *in vivo*. Prior metformin exposure *in vivo* also enhanced GC proliferation *in vitro.* GCs from patients with PCOS consistently had a significantly reduced capacity to synthesize progesterone compared with controls. These results significantly advance the existing knowledge on the perturbance of glucose metabolism by the GCs in women with PCOS by revealing reduced pyruvate production and preferential lactate production by these cells in addition to their reduced glucose uptake. This metabolic lesion may compromise the availability of GC-derived pyruvate, the key energy substrate for oocyte development and maturation, and so negatively impact on oocyte quality. The findings that metformin pretreatment *in vivo* improved acute glucose consumption and utilization by GCs and caused a reduction in long-term progesterone production by GCs are novel. Further research is required to underpin the molecular mechanisms of the effect(s) of metformin on GCs in order to optimize the clinical use of this drug.

## Authors' roles

D.M. executed the research, analysed the data and drafted the manuscript. S.E.H. assisted with GC cultures and validation of GC metabolism assays. A.H.B. supervised the clinical aspects of the research and revised the manuscript. J.H.B. conducted the insulin and glucose assays in serum and follicular fluid. B.K.C. conducted the progesterone assays at the laboratory of Nottingham University. H.M.P. designed the experimental strategy and research methodology, and supported data analysis and the drafting of the manuscript.

## Funding

This work was supported by the UK Medical Research Council Grant Reference number G0800250. Funding to pay the Open Access publication charges for this article was provided by the University of Leeds, Accounts Payable, EC Stoner Building, Leeds, LS2 9JT, UK.

## Accessing Research Data

Data can be accessed via email by contacting H.M.Picton@leeds.ac.uk

## Conflict of interest

None declared.

## References

[DEU187C1] Attia GR, Rainey WE, Carr BR (2001). Metformin directly inhibits androgen production in human thecal cells. Fertil Steril.

[DEU187C2] Berker B, Emral R, Demirel C, Corapcioglu D, Unlu C, Kose K (2004). Increased insulin-like growth factor-i levels in women with polycystic ovary syndrome, and beneficial effects of metformin therapy. Gynecol Endocrinol.

[DEU187C3] Billig H, Hedin L, Magnusson C (1983). Gonadotropins stimulate lactate production by rat cumulus and granulosa-cells. Acta Endocrinol.

[DEU187C4] Binoux M (1995). The IGF system in metabolism regulation. Diabetes Metab.

[DEU187C5] Boland NI, Humpherson PG, Leese HJ, Gosden RG (1993). Pattern of lactate production and steroidogenesis during growth and maturation of mouse ovarian follicles in vitro. Biol Reprod.

[DEU187C6] Boland NI, Humpherson PG, Leese HJ, Gosden RG (1994). Characterization of follicular energy metabolism. Hum Reprod.

[DEU187C7] Book CB, Dunaif A (1999). Selective insulin resistance in the polycystic ovary syndrome. J Clin Endocrinol Metab.

[DEU187C8] Breckwoldt M, Selvaraj N, Aharoni D, Barash A, Segal I, Insler V, Amsterdam A (1996). Expression of ad4-bp/cytochrome p450 side chain cleavage enzyme and induction of cell death in long-term cultures of human granulosa cells. Mol Hum Reprod.

[DEU187C9] Campbell BK, Scaramuzzi RJ, Webb R (1996). Induction and maintenance of oestradiol and immunoreactive inhibin production with FSH by ovine granulosa cells cultured in serum-free media. J Reprod Fertil.

[DEU187C10] Campbell BK, Baird DT, Webb R (1998). Effects of dose of LH on androgen production and luteinization of ovine theca cells cultured in a serum-free system. J Reprod Fertil.

[DEU187C11] Campbell BK, Onions V, Kendall NR, Guo L, Scaramuzzi RJ (2010). The effect of monosaccharide sugars and pyruvate on the differentiation and metabolism of sheep granulosa cells *in vitro*. Reproduction.

[DEU187C12] Ciaraldi TP, Morales AJ, Hickman MG, Odom-Ford R, Olefsky JM, Yen SS (1997). Cellular insulin resistance in adipocytes from obese polycystic ovary syndrome subjects involves adenosine modulation of insulin sensitivity. J Clin Endocrinol Metab.

[DEU187C13] Corbould A, Kim YB, Youngren JF, Pender C, Kahn BB, Lee A, Dunaif A (2005). Insulin resistance in the skeletal muscle of women with PCOS involves intrinsic and acquired defects in insulin signaling. Am J Physiol Endocrinol Metab.

[DEU187C14] Cusi K, DeFronzo RA (1998). Metformin: a review of its metabolic effects. Diabetes Rev.

[DEU187C15] Das M, Djahanbakhch O, Hacihanefioglu B, Saridogan E, Ikram M, Ghali L, Raveendran M, Storey A (2008). Granulosa cell survival and proliferation are altered in polycystic ovary syndrome. J Clin Endocrinol Metab.

[DEU187C16] Doldi N, Gessi A, Destefani A, Calzi F, Ferrari A (1998). Polycystic ovary syndrome: anomalies in progesterone production. Hum Reprod.

[DEU187C17] Dominguez LJ, Davidoff AJ, Srinivas PR, Standley PR, Walsh MF, Sowers JR (1996). Effects of metformin on tyrosine kinase activity, glucose transport, and intracellular calcium in rat vascular smooth muscle. Endocrinology.

[DEU187C18] Dunaif A (1997). Insulin resistance and the polycystic ovary syndrome: mechanism and implications for pathogenesis. Endocr Rev.

[DEU187C19] Dunaif A (2006). Insulin resistance in women with polycystic ovary syndrome. Fertil Steril.

[DEU187C20] Erickson GF, Magoffin DA, Garzo VG, Cheung AP, Chang RJ (1992). Granulosa cells of polycystic ovaries: are they normal or abnormal?. Hum Reprod.

[DEU187C21] ESHRE/ASRM TR (2004). Revised 2003 consensus on diagnostic criteria and long-term health risks related to polycystic ovary syndrome (PCOS). Hum Reprod.

[DEU187C22] Fedorcsak P, Storeng R, Dale PO, Tanbo T, Abyholm T (2000). Impaired insulin action on granulosa-lutein cells in women with polycystic ovary syndrome and insulin resistance. Gynecol Endocrinol.

[DEU187C23] Fleming R, McQueen D, Yates RWS, Coutts JRT (1995). Spontaneous follicular and luteal function in infertile women with oligomenorrhoea: role of luteinizing hormone. Clin Endocrinol.

[DEU187C24] Fryer LGD, Parbu-Patel A, Carling D (2002). The anti-diabetic drugs rosiglitazone and metformin stimulate amp-activated protein kinase through distinct signaling pathways. J Biol Chem.

[DEU187C25] Fulgencio JP, Kohl C, Girard J, Pegorier JP (2001). Effect of metformin on fatty acid and glucose metabolism in freshly isolated hepatocytes and on specific gene expression in cultured hepatocytes. Biochem Pharmacol.

[DEU187C26] Girard J, Postic C, Burcelin R, Guillet I, Leturque A (1992). Glucose transporters. Physiology and physiopathology. Presse Med.

[DEU187C27] Gunton JE, Delhanty PJD, Takahashi SI, Baxter RC (2003). Metformin rapidly increases insulin receptor activation in human liver and signals preferentially through insulin-receptor substrate-2. J Clin Endocrinol Metab.

[DEU187C28] Harlow CR, Winston RML, Margara RA, Hillier SG (1987). Gonadotropic control of human granulosa-cell glycolysis. Hum Reprod.

[DEU187C29] Harris SE, Adriaens I, Leese HJ, Gosden RG, Picton HM (2007). Carbohydrate metabolism by murine ovarian follicles and oocytes grown in vitro. Reproduction.

[DEU187C30] Harris SE, Leese HJ, Gosden RG, Picton HM (2009). Pyruvate and oxygen consumption throughout the growth and development of murine oocytes. Mol Reprod Dev.

[DEU187C31] Harris SE, Maruthini D, Tang T, Balen AH, Picton HM (2010). Metabolism and karyotype analysis of oocytes from patients with polycystic ovary syndrome. Hum Reprod.

[DEU187C32] Havelock JC, Rainey WE, Carr BR (2004). Ovarian granulosa cell lines. Mol Cell Endocrinol.

[DEU187C33] Hennessy BT, Smith DL, Ram PT, Lu YL, Mills GB (2005). Exploiting the pi3k/akt pathway for cancer drug discovery. Nat Rev Drug Discov.

[DEU187C34] Hugentobler SA, Humpherson PG, Leese HJ, Sreenan JM, Morris DG (2008). Energy substrates in bovine oviduct and uterine fluid and blood plasma during the oestrous cycle. Mol Reprod Dev.

[DEU187C35] Jalving M, Gietema JA, Lefrandt JD, de Jong S, Reyners AKL, Gans ROB, De Vries EGE (2010). Metformin: taking away the candy for cancer?. Eur J Cancer.

[DEU187C36] Joseph-Horne R, Mason H, Batty S, White D, Hillier S, Urquhart M, Franks S (2002). Luteal phase progesterone excretion in ovulatory women with polycystic ovaries. Hum Reprod.

[DEU187C37] Kausch C, Krutzfeldt J, Witke A, Rettig A, Bachmann O, Rett K, Matthaei S, Machicao F, Haring HU, Stumvoll M (2001). Effects of troglitazone on cellular differentiation, insulin signaling, and glucose metabolism in cultured human skeletal muscle cells. Biochem Biophys Res Commun.

[DEU187C38] la Marca A, Morgante G, Palumbo M, Cianci A, Petraglia F, De Leo V (2002). Insulin-lowering treatment reduces aromatase activity in response to follicle-stimulating hormone in women with polycystic ovary syndrome. Fertil Steril.

[DEU187C39] Lee MS, Kim SH, Kim DS, Min KS, Yoon JT (2012). Metformin enhances the action of insulin on porcine granulosa-lutein cells *in vitro*. Anim Reprod Sci.

[DEU187C40] Leese HJ, Lenton EA (1990). Glucose and lactate in human follicular fluid: concentrations and interrelationships. Hum Reprod.

[DEU187C41] Lieberman M, Marks A, Liebermann M, Marks A (2009). Basic concepts in the regulation of fuel metabolism by insulin, glucagon, and other hormones. Marks’ Basic Medical Biochemistry—A Clinical Approach.

[DEU187C42] Lin Y, Fridstrom M, Hillensjo T (1997). Insulin stimulation of lactate accumulation in isolated human granulosa-luteal cells: a comparison between normal and polycystic ovaries. Hum Reprod.

[DEU187C43] Lopaczynski W (1999). Differential regulation of signaling pathways for insulin and insulin-like growth factor I. Acta Biochim Pol.

[DEU187C44] Mansfield R, Galea R, Brincat M, Hole D, Mason H (2003). Metformin has direct effects on human ovarian steroidogenesis. Fertil Steril.

[DEU187C45] Mather KJ, Hunt AE, Steinberg HO, Paradisi G, Hook G, Katz A, Quon MJ, Baron AD (2001). Repeatability characteristics of simple indices of insulin resistance: implications for research applications. J Clin Endocrinol Metab.

[DEU187C46] Meenakumari KJ, Agarwal S, Krishna A, Pandey LK (2004). Effects of metformin treatment on luteal phase progesterone concentration in polycystic ovary syndrome. Braz J Med Biol Res.

[DEU187C47] Moghetti P, Castello R, Negri C, Tosi F, Perrone F, Caputo M, Zanolin E, Muggeo M (2000). Metformin effects on clinical features, endocrine and metabolic profiles, and insulin sensitivity in polycystic ovary syndrome: a randomized, double-blind, placebo-controlled 6-month trial, followed by open, long-term clinical evaluation. J Clin Endocrinol Metab.

[DEU187C48] Musi N, Hirshman MF, Nygren J, Svanfeldt M, Bavenholm P, Rooyackers O, Zhou GC, Williamson JM, Ljunqvist O, Efendic S (2002). Metformin increases AMP-activated protein kinase activity in skeletal muscle of subjects with type 2 diabetes. Diabetes.

[DEU187C49] Nafiye Y, Sevtap K, Muammer D, Emre O, Senol K, Leyla M (2010). The effect of serum and intrafollicular insulin resistance parameters and homocysteine levels of nonobese, nonhyperandrogenemic polycystic ovary syndrome patients on in vitro fertilization outcome. Fertil Steril.

[DEU187C50] Nestler JE, Jakubowicz DJ (1997). Lean women with polycystic ovary syndrome respond to insulin reduction with decreases in ovarian p450c17 alpha activity and serum androgens. J Clin Endocrinol Metab.

[DEU187C51] Palomba S, Falbo A, Russo T, Orio F, Tolino A, Zullo F (2010). Systemic and local effects of metformin administration in patients with polycystic ovary syndrome (PCOS): relationship to the ovulatory response. Hum Reprod.

[DEU187C52a] Palomba S, Falbo A, La Sala GB (2013). Effects of metformin in women with polycystic ovary syndrome treated with gonadotrophins for *in vitro* fertilisation and intracytoplasmic sperm injection cycles: A systematic review and meta-analysis of randomised controlled trials. BJOG.

[DEU187C52] Palomba S, Falbo A, La Sala GB (2014). Metformin and gonadotropins for ovulation induction in patients with polycystic ovary syndrome: a systematic review with meta-analysis of randomized controlled trials. Reprod Biol Endocrinol.

[DEU187C53] Pasquali R, Gambineri A (2009). Targeting insulin sensitivity in the treatment of polycystic ovary syndrome. Expert Opin Ther Targets.

[DEU187C54] Phy JL, Conover CA, Abbott DH, Zschunke MA, Walker DL, Session DR, Tummon IS, Thornhill AR, Lesnick TG, Dumesic DA (2004). Insulin and messenger ribonucleic acid expression of insulin receptor isoforms in ovarian follicles from nonhirsute ovulatory women and polycystic ovary syndrome patients. J Clin Endocrinol Metab.

[DEU187C55] Picton HM, Campbell BK, Hunter MG (1999). Maintenance of oestradiol production and expression of cytochrome p450 aromatase enzyme mRNA in long-term serum-free cultures of pig granulosa cells. J Reprod Fertil.

[DEU187C56] Poretsky L, Seto-Young D, Shrestha A, Dhillon S, Mirjany M, Liu HC, Yih MC, Rosenwaks Z (2001). Phosphatidyl-inositol-3 kinase-independent insulin action pathway(s) in the human ovary. J Clin Endocrinol Metab.

[DEU187C57] Prelevic GM (1997). Insulin resistance in polycystic ovary syndrome. Curr Opin Obstet Gynecol.

[DEU187C58] Purcell SH, Chi MM, Lanzendorf S, Moley KH (2012). Insulin-stimulated glucose uptake occurs in specialized cells within the cumulus oocyte complex. Endocrinology.

[DEU187C59] Rice S, Christoforidis N, Gadd C, Nikolaou D, Seyani L, Donaldson A, Margara R, Hardy K, Franks S (2005). Impaired insulin-dependent glucose metabolism in granulosa-lutein cells from anovulatory women with polycystic ovaries. Hum Reprod.

[DEU187C60] Robert F, Fendri S, Hary L, Lacroix C, Andrejak M, Lalau JD (2003). Kinetics of plasma and erythrocyte metformin after acute administration in healthy subjects. Diabetes Metab.

[DEU187C61] Santana LF, Fernandez-Santoss JM, Hernaez MJ, Caligara C, Reis RM, Fernandez-Sanchez M (2007). Gene expression of IGF-1 receptor in human luteinized granulosa cumulus cells from non obese and non insulin resistant women with polycystic ovary syndrome (PCOS) with and without metformin treatment. Fertil Steril.

[DEU187C62] Santos RF, Nomizo R, Bopsco A, Wajchenberg BL, Reaven GM, Azhar S (1997). Effect of metformin on insulin-stimulated tyrosine kinase activity of erythrocytes from obese women with normal glucose tolerance. Diabetes Metab.

[DEU187C63] Sarabia V, Lam L, Burdett E, Leiter LA, Klip A (1992). Glucose-transport in human skeletal-muscle cells in culture—stimulation by insulin and metformin. J Clin Invest.

[DEU187C64] Scheen AJ (1996). Clinical pharmacokinetics of metformin. Clin Pharmacokinet.

[DEU187C65] Scheepers A, Joost HG, Schurmann A (2004). The glucose transporter families SGLT and GLUT: molecular basis of normal and aberrant function. JPEN J Parenter Enteral Nutr.

[DEU187C66] Seto-Young D, Zajac J, Liu HC, Rosenwaks Z, Poretsky L (2003). The role of mitogen-activated protein kinase in insulin and insulin-like growth factor I (IGF-I) signaling cascades for progesterone and IGF-binding protein-1 production in human granulosa cells. J Clin Endocrinol Metab.

[DEU187C67] Shulman GI (2000). Cellular mechanisms of insulin resistance. J Clin Invest.

[DEU187C68] Sonntag B, Gotte M, Wulfing P, Schuring AN, Kiesel L, Greb RR (2005). Metformin alters insulin signaling and viability of human granulosa cells. Fertil Steril.

[DEU187C69] Stadtmauer LA, Toma SK, Riehl RM, Talbert LM (2001). Metformin treatment of patients with polycystic ovary syndrome undergoing *in vitro* fertilization improves outcomes and is associated with modulation of the insulin-like growth factors. Fertil Steril.

[DEU187C70] Sutton-McDowall ML, Gilchrist RB, Thompson JG (2010). The pivotal role of glucose metabolism in determining oocyte developmental competence. Reproduction.

[DEU187C71] Takata K (1996). Glucose transporters in the transepithelial transport of glucose. J Electron Microsc (Tokyo).

[DEU187C72] Tang T, Glanville J, Hayden CJ, White D, Barth JH, Balen AH (2006a). Combined lifestyle modification and metformin in obese patients with polycystic ovary syndrome. A randomized, placebo-controlled, double-blind multicentre study. Hum Reprod.

[DEU187C73] Tang T, Glanville J, Orsi N, Barth JH, Balen AH (2006b). The use of metformin for women with PCOS undergoing IVF treatment. Hum Reprod.

[DEU187C74] Tosca L, Solnais P, Ferre P, Foufelle F, Dupont J (2006). Metformin-induced stimulation of adenosine 5′ monophosphate-activated protein kinase (PRKA) impairs progesterone secretion in rat granulosa cells. Biol Reprod.

[DEU187C75] Tosca L, Chabrolle C, Uzbekova S, Dupont J (2007). Effects of metformin on bovine granulosa cells steroidogenesis: Possible involvement of adenosine 5′ monophosphate-activated protein kinase (AMPK). Biol Reprod.

[DEU187C76] Unfer V, Casini ML, Marelli G, Costabile L, Gerli S, Di Renzo GC (2005). Different routes of progesterone administration and polycystic ovary syndrome: a review of the literature. Gynecol Endocrinol.

[DEU187C77] Venkatesan AM, Dunaif A, Corbould A (2001). Insulin resistance in polycystic ovary syndrome: progress and paradoxes. Recent Prog Horm Res.

[DEU187C78] Wiernsperger NF, Bailey CJ (1999). The antihyperglycaemic effect of metformin—therapeutic and cellular mechanisms. Drugs.

[DEU187C79] Wiholm BE, Myrhed M (1993). Metformin-associated lactic-acidosis in Sweden 1977–1991. Eur J Clin Pharmacol.

[DEU187C80] Wijeyaratne CN, Balen AH, Barth JH, Belchetz PE (2002). Clinical manifestations and insulin resistance (IR) in polycystic ovary syndrome (PCOS) among South Asians and Caucasians: is there a difference?. Clin Endocrinol (Oxf).

[DEU187C81] Wild S, Pierpoint T, Jacobs H, McKeigue P (2000). Long-term consequences of polycystic ovary syndrome: results of a 31 year follow-up study. Hum Fertil (Camb).

[DEU187C82] Willis D, Franks S (1995). Insulin action in human granulosa cells from normal and polycystic ovaries is mediated by the insulin receptor and not the type-I insulin-like growth factor receptor. J Clin Endocrinol Metab.

[DEU187C83] Wright AD, Cull CA, Holman RR, Turner RC (1998). UKPDS 28: a randomized trial of efficacy of early addition of metformin in sulfonylurea-treated type 2 diabetes. Diabetes Care.

[DEU187C84] Wu XK, Zhou SY, Liu JX, Pollanen P, Sallinen K, Makinen M, Erkkola R (2003). Selective ovary resistance to insulin signaling in women with polycystic ovary syndrome. Fertil Steril.

